# Implications of Endogenous Small Regulatory RNAs Survey in Mollusks on Gene Silencing Approaches

**DOI:** 10.1007/s10126-025-10519-9

**Published:** 2025-09-29

**Authors:** Cory Von Eiff, Beatriz Schueng Zancanela, Megan Gima, Kevin Quito, Manitejus Kotikalapudi, Sergio Valdivia, Yulica Santos-Ortega, Alex Sutton Flynt

**Affiliations:** 1 School of Biological Environmental and Earth Sciences, University of Southern Mississippi, Hattiesburg, MS 39406 USA; 2 Department of Biomedical Engineering, University of Mississippi, Brevard Hall D303, Oxford, MS 38677 USA; 3 Office of the Vice President for Research, Thad Cochran Marine Aquaculture Center, University of Southern Mississippi, Ocean Springs, MS 39564 USA; 4 MD Anderson Cancer Biology Department, UT Health MD Anderson GSBS, Houston, TX 77030 USA

**Keywords:** Mollusks, RNAi, sRNAs, piRNAs, Lophotrochozoa

## Abstract

Mollusks are an abundant group of animals, with many ecologically and economically important members that are phylogenetically distinct from nearly all genetic model organisms. This study provides a clade-wide evaluation of small RNA biogenesis pathways, with emphasis on the eastern oyster, *Crassostrea virginica*. A more thorough characterization of these molecules supports rationale design of RNA interference (RNAi) approaches for manipulation of mollusk genetics. Like other animal groups, mollusks have conserved microRNAs (miRNAs), with some shared with ecdysozoans and deuterostomes; however, there was no evidence of a dedicated endogenous small-interfering RNA (siRNA) pathway. These findings suggest that alternatives to long double-stranded RNA (dsRNA)-mediated knockdown, specifically short-hairpin RNAs or small duplex RNAs, are likely more appropriate for gene silencing in mollusks. The study also finds abundant Piwi-interacting RNAs (piRNAs) in both soma and gonads with some mollusk-specific aspects. Many invertebrates exhibit somatic piRNAs; however, mollusk piRNAs appear to be restricted to a subset of cells, suggesting that the potential of piRNA-based RNAi is also limited. Further, individual animals also express a unique collection of piRNAs that seem to be only partially determined through inheritance from parents. Together, this work defines the RNAi mechanisms in mollusks and provides insights into the phenotypic diversity seen in this group.

**Supplementary Information:**

The online version contains supplementary material available at 10.1007/s10126-025-10519-9.

## Significance Statement

This study provides an extensive, clade-wide evaluation of small RNA (sRNA) biogenesis pathways in mollusks. Our findings reveal that, unlike ecdysozoans, mollusks lack a dedicated siRNA pathway, which influences design of RNA interference (RNAi) applications in mollusks. Instead, we find the expected microRNAs (miRNAs) and an assortment of Piwi-interacting RNAs (piRNAs). We show that piRNA biology in mollusks is highly cell-type specific and genetically individualized. We further demonstrate that piRNA generation is likely restricted to stem-like cells, suggesting a role in genomic maintenance in this cell subset. This work offers insight into RNAi mechanisms in mollusks, the second largest animal phylum, and has significant implications for both genetic investigation and applications for pest control and aquaculture.

## Introduction

Mollusks are an abundant and diverse group of animals, with many ecologically and economically important members that are the basis of major fisheries. An average of $20.6 billion dollars of economic activity from 2010 to 2015 was associated with mollusk fisheries, accounting for 14% of total marine fisheries (Wijsman et al. 2019). Approximately 90% of the bivalve harvest was from aquaculture. As there is extensive human interaction with these animals, there is an opportunity to integrate biotechnologies into husbandry practices to enhance yields. Possibly the most facile genetic technology to deploy is RNA interference (RNAi) due to the simple nature of double-stranded RNAs (dsRNAs) typically used to trigger the process and elicit gene silencing. In animals, three RNAi pathways have been described: microRNAs (miRNAs), small interfering RNAs (siRNAs), and Piwi-interacting RNAs (piRNAs) (Großhans and Filipowicz 2008; Iwasaki et al. 2015; Ozata et al. 2019). Each pathway has been used for gene silencing through leveraging distinct biogenesis mechanisms to guide exogenous RNAs into respective effector Argonaute (Ago)/Piwi proteins (Farazi et al. 2008; Carthew and Sontheimer 2009; Watanabe and Lin 2014). Differences in these pathways are often observed at the order level but have even been seen between species (Flynt 2021; Khanal et al. 2022). Thus, an understanding of a target species’ endogenous small RNA biology may facilitate choosing an approach most likely to elicit successful RNAi-mediated gene silencing (Flynt 2021).

Among all major animal groups, sRNA pathways have been relatively unexplored in the Mollusca phylum and the broader lophotrochozoan clade (Jehn et al. 2018). Of these pathways, miRNAs are the most conserved, being ubiquitously identified in eukaryotes, including mollusks (Feng et al. 2020). Originating from “short” (~ 70 nt) hairpins, miRNAs are cropped by Drosha and cleaved by a miRNA processing Dicer (miDicer) (Lee et al. 2003; Lund et al. 2004; Bartel 2004; Huang et al. 2021). Both Drosha and Dicer are RNase III class enzymes that leave 2nt 3’ overhangs on small RNA duplex products (Ghosh et al. 2009; Kim et al. 2017). After processing, miRNAs load into miRNA-specific Ago (miAgo) proteins and initiate degradation of mRNA by inhibiting translation or occasionally directing cleavage of the bound RNA, thus regulating gene expression (Valencia-Sanchez et al. 2006; Feng et al. 2020). In contrast, siRNAs, which are reported in many ecdysozoans, originate from long double-stranded RNA (dsRNA), and after processing by a dedicated siRNA generating Dicer (siDicer), interact with siRNA-specific AGO (siAGO) proteins to initiate target cleavage. Often, siRNAs have anti-viral function, though there are many endogenous siRNAs (endo-siRNAs) that have cryptic roles that include genome maintenance and gene regulatory networks (Lee et al. 2004; Mello and Jr 2004; Okamura and Lai 2008; Carthew and Sontheimer 2009; Gammon and Mello 2015). Endo-siRNAs are poorly conserved, being mostly absent in vertebrates and the annelid *Capitella teleta* (Wynant et al. 2017; Huang et al. 2021; Khanal et al. 2022).

The third class, piRNAs, are present in nearly all animals, including in mollusks, with a notable exception of dust mites and some platyhelminths (Jehn et al. 2018; Mondal et al. 2018; Fontenla et al. 2021). Unlike miRNAs and siRNAs, piRNAs are not derived from dsRNA precursors (Aravin et al. 2007; Zamore 2010). Lack of Dicer processing and differences between the small RNA binding pocket of Ago and Piwi proteins lead to piRNAs having a distinct size of ~ 26–30 nt (Cheng et al. 2019; Yamaguchi et al. 2020). piRNAs originate through two mechanisms: phasing and Ping Pong (Senti and Brennecke 2010). Both pathways involve the interplay of Piwi partner proteins, which we define here as phasing Piwi (phPiwi) and responder Piwi (rePiwi) based on their respective role in phasing piRNA biogenesis. In phasing, a responder piRNA directs cleavage of a transcript that becomes a substrate for the Zucchini/mito-PLD (Zuc) RNase (Zamore 2010; Nishimasu et al. 2012; Zhang et al. 2023). Zuc processing yields a distinctive head-to-tail arrangement, thus the designation as “phasing” piRNAs. Cleavage usually occurs at uridine residues, leading to 1 U bias of phasing piRNAs. Using these features, phasing piRNAs can be recognized in small RNA sequencing alignments when reads show 1U and are arranged end-to-end (Brennecke et al. 2008; Klattenhoff and Theurkauf 2008; Sun et al. 2020). Ping Pong piRNAs are the result of an amplification loop coordinated by phPiwi and rePiwi. Transcripts cleaved by phPiwi become precursors of rePiwi-bound piRNAs and vice versa, leading to amplifying piRNA production (Czech and Hannon 2016; Czech et al. 2018). piRNAs generated by Ping Pong have a characteristic 10 nt overlap, which can be observed in read alignments (Iwasaki et al. 2015). piRNAs have been found to silence transposable elements and mRNAs in a variety of animals (Luo and Lu 2017; Praher et al. 2017; Larriba and Del Mazo 2018; Lewis et al. 2018; Mondal et al. 2020; van Lopik et al. 2023). They are expressed in both somatic and gonadal tissue of mollusks (Jehn et al. 2018; Huang et al. 2019).

RNAi-mediated gene silencing has been reported in fewer than two dozen mollusk species (Korneev et al. 2002; Jiang et al. 2006; Fabioux et al. 2009; Suzuki et al. 2009; Fang et al. 2011; Knight et al. 2011; Lu and Feng 2011; Huvet et al. 2012, 2015; Choi et al. 2013; Owens and Malham 2015; Masood et al. 2016, 2017; Miao et al. 2016; Yu et al. 2016; Zhao et al. 2016; Ma et al. 2017; Feng et al. 2019; Liu et al. 2019; Zheng et al. 2022). Most attempts involved soaking or injection of long synthetic dsRNAs, with the intention of these molecules being processed into siRNAs that guide silencing of targets, as occurs in ecdysozoans like *D. melanogaster* and *C. elegans* as well as the planarian, *Schmidtea mediterranea*. While work in planarians would suggest that mollusks can silence genes through siRNAs processed from long dsRNA, further work in *C. teleta* (an annelid) shows other animals in Spiralia do not make observable endo-siRNAs (Khanal et al. 2022). Within Spiralia, platyhelminths are basal to annelids and mollusks (Struck et al. 2014; Mousavi et al. 2019). Consequently, if loss of a dedicated siRNA pathway occurred before the split of annelids and mollusks, then both groups may not have endo-siRNAs. This motivates a re-evaluation of long dsRNA-based gene silencing in mollusks. To establish approaches for gene silencing in these animals, we conducted a survey of the sRNAs found in mollusks. We investigated sRNA biology in 32 mollusks, with emphasis on the eastern oyster, *Crassostrea virginica*. Results show significant similarities between annelids and mollusks. Both clearly have miRNAs and piRNAs, but there is no evidence of endo-siRNAs. This suggests that alternatives to long dsRNA may be more effective at triggering RNAi in mollusks. Investigation of the other pathways, miRNAs and piRNAs, found that while miRNAs function similar to other animals' miRNAs, piRNAs appear to be restricted to specific cell types while also showing a high level of variability among individuals.

## Materials and Methods

### Animal Husbandry

*C. virginica* specimens were obtained from the Thad Cochran Marine Aquaculture Center in Ocean Springs, Mississippi. Specimens were kept in a 200-gallon tank with biofilters and a UV sterilizer, and we maintained ~ 20 ppt salinity and regularly monitored levels of pH, ammonia, nitrate, and nitrite. For feeding, we moved the oysters into a separate container with sea water and fed them ~ 30 mL (~ 6 × 10^10^ algae cells) of Shellfish Diet © 1800 every other day for ~ 4 h each feeding.

*C. teleta* worms were obtained from the lab of Dr. Elaine Seaver at the Whitney Laboratory for Marine Bioscience at the University of Florida. Housed in a 20 °C growth chamber, our specimens were kept inside containers with 100 ml of estuary mud and 200 ml of salt water. Each container held ~ 20 worms, evenly divided between males and females. The boxes were monitored for the presence of swimming larva, which were then transported to new containers. The worms were fed weekly by additional estuarine mud.

*L. variegatus* specimens were obtained from Gulf Specimen Marine Lab in Panacea, Florida. Animals were kept in a 50-gallon tank with biofilters and large rocks to simulate the conditions of a reef, with ~ 25 ppt salinity. The urchins were fed every other day one-third of dried nori algae sheets bought from a local market.

A female *O. bimaculoides* was obtained from Marine Biological Laboratory. The octopus was housed in a 70-gallon tank with biofilters and ~ 36 ppt salinity. A chiller was attached to the pump to keep the water at ~ 18 °C. Clay pots, rocks of varying sizes, and plastic blocks were placed inside the tank for shelter and entertainment. The octopus was fed thawed shrimp daily.

A live specimen of *H. rufescens* (red abalone) was purchased from Monterey Abalone Company and sacrificed immediately to acquire samples.

### Animal Injury and Regeneration

#### Crassostrea virginica

Three adult oysters of similar size were selected and injured by first shaving their shell to create a smooth surface followed by drilling a hole through the shell into the Mantle region. The oysters were allowed 2 weeks to partially regenerate their shells, which we confirmed by observing a thin layer of secreted shell covering the drilled holes. After 2 weeks, we extracted proximal mantle tissue directly underneath the injury site to serve as the regenerative sample. We also collected distal tissue from the opposite side of the mantle to serve as the control. This protocol was based on a similar experiment that observed miRNA and gene expression in repair and biomineralization in freshwater snails (Cerveau and Jackson 2021).

#### Capitella teleta

Worms were placed under a dissecting microscope and amputated at the 12th body segment using forceps. They were then placed in 20 mL of filtered salt water and allowed to recover for 24–48 h. After the recovery period, they were placed in containers with mud and salt water. The worms were checked every other day for growth and survivability, and the regenerated tissue was collected after 2–3 weeks.

#### Lytechnius variegatus

For urchin amputations, each individual was injured using a pair of fine scissors and nail clippers; about half of the urchin’s spines were cut very close to the test (hard shell). Once the tube feet were exposed, a pair of fine scissors was used to cut the tube feet. The urchins were placed back into the tank and allowed to regenerate for 1 week. After they had sufficient time to regrow some of the tube feet, they were placed back into smaller containers, and the process was repeated. The previously injured half of the urchin was collected from the container using a pipette and strained using a 40 µm cell strainer to remove salt water and retain the tube feet. The unharmed half of the urchin went through the same process and was used as control.

#### Octopus bimaculoides

The octopus used in this study was handled following ethical considerations established by the European Parliament and the Council of the European Union. Before injury, it was anaesthetized using ethanol. 0.25% increments v/v of ethanol were added every 2 min to a holding container until a final concentration of 1.5% EtOH was reached. After the animal was under complete anesthesia for ~ 14 min, the tips (~ 2 cm) of three tentacles were amputated using a sterilized razor blade and collected as control samples. After allowing the tentacles to regenerate for three days, the above protocol was repeated to collect the blastemas that had formed. The animal was euthanized using the same approach as anesthesia until the EtOH concentration had reached a lethal concentration.

### Breeding and Offspring Collection

A male and two female oysters were exposed to heated seawater (32 °C) to induce gamete release. These parental animals were sacrificed and their muscle, mantle, gill, and gonad tissues collected. Eggs from each female were fertilized with sperm from the male, with the two crosses cultured separately. The resulting embryos from each cross were cultured to the pediveliger stage, harvested, and set on microculture to create single set oysters. Post-set (seed) were cultured in upwelling nursery systems for ~ 3 months. Several of the offspring from each of the two crosses were sacrificed and their entire bodies used for RNA extraction.

### Animal Rights Statement

All animals studied were invertebrates that were analyzed in a laboratory setting. No specimens were released into the environment. Animals were treated with best practices to minimize suffering when appropriate.

### RNA Extraction and Sequencing

All harvested tissues were placed in 700 µL of TRizol LS and 300 µL of nuclease free water. Addition of water was necessary to balance excess salts present in the tissues of these Marine animals to make samples compatible with the TRizol method. After tissue homogenization, samples were processed following manufacturer protocols and the resulting isolated RNA were resuspended in 30 µL of nuclease free water. Total RNA samples were sent to the University of Mississippi Medical Center Genome Core, which prepared libraries using the Illumina small RNA TruSeq library construction kit and using an Illumina NextSeq2000.

### Phylogenetic Analysis

We searched NCBI for Dicer and Ago/Piwi annotations in Mollusca species. After identifying numerous annotations, we further searched for homologs using Ensembl Metazoa and NCBI BLAST searches. We then imported our resulting Dicer and Ago/Piwi protein sequences into Molecular Evolutionary Genetic Analysis Version 12 (MEGA12), which utilizes Multiple Sequence Comparison by Log-Expectation (MUSCLE) for phylogenetic analysis.

### Small RNA Analysis Pipeline

The following genome sequences and annotation files were acquired from NCBI’s GenBank database: *C. virginica* (GCA_002022765.4), *Magallana gigas* (GCA_963853765.1), *Haliotis rufescens* (GCA_023055435.1), *Lytechinus variegatus* (GCA_018143015.1), *Octopus bimaculoides* (GCA_001194135.2), *Rapana venosa* (GCA_028751875.1), and *Lymnaea stagnalis* (GCA_900036025.1). Genome sequence and genome annotation files for *C. teleta* were acquired from Ensembl Metazoa (Capitella_teleta_v1.0). We clipped the adaptors off the resulting high-throughput sequencing data using CutAdapt and then used bowtie and samtools to map and process alignments to respective genomes. Using a python-based algorithm, we screened for 15−30nt sRNAs that had 2 nt overlaps (indicating Dicer signature found in siRNAs) or 10 nt overlaps (indicating Ping Pong signature found in piRNAs) (Antoniewski 2014). piRNA loci were analyzed for phasing using an approach described by the piPipes (Han et al. 2015). We also generated a read counts table that could be visualized using ggplot2 and pheatmap. DESeq2 was used to compare the piRNA expression of individual intact tissues to one another.

### In Situ Hybridization and Single-Nuclei Sequencing

To localize *C. virginica* Piwis, we harvested mantle, gill, gonad, and muscle tissue. After freezing the extracted tissues in OCT compound, we used a cryostat to section and mount 5nm tissue on Plus Gold slides. To generate a phasPiwi probe, we first cloned a portion of phasPiwi via PCR using phasPiwi-specific primers (Forward: ATGTCAGGTAGAGGAAGAGCTCGTG; Reverse: TCAAGCTATGCATCCAACGCGTTGGGTTTGTTCACATGTCCAAGCTTGC). The ThermoFisher Phire Hot Start II kit was used, and the samples cycled 35 times, with denaturation for 5 s at 98 °C, annealing for 15 s at 60 °C, and denaturation for 60 s at 72 °C. Following PCR amplification, T7 in vitro transcription was used to create a probe labeled with Digoxygenin. We then performed in situ hybridization as previously described (Barthel and Raymond 1993). For single-nuclei sequencing, mantle tissue freshly embedded in OCT was sent to the genomics core at the UMMC for single-nuclei sequencing using a Chromium X with the Chromium Next GEM Single Cell 3' Kit v3.1 followed by sequencing on an Illumina NextSeq 2000.

## Results

### Conservation of RNAi Machinery in Mollusks

**Fig. 1:**
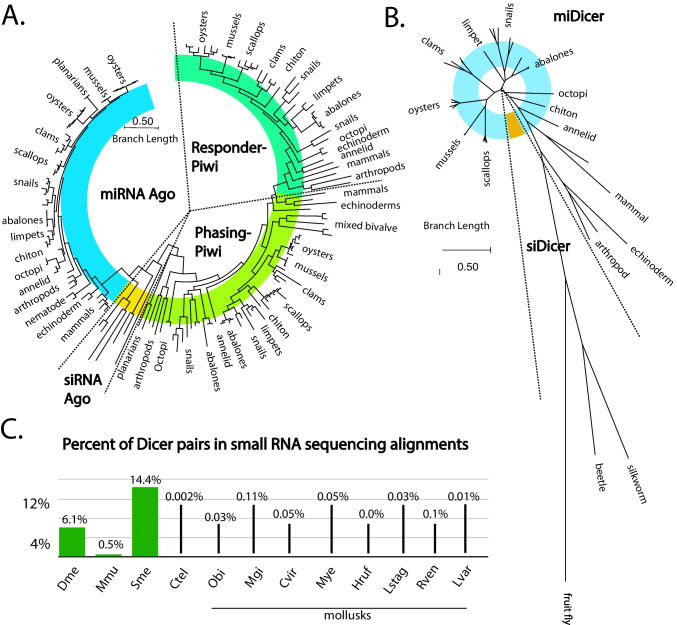
Argonaute, Dicer, and siRNA presence in Mollusca. **A** Overview of phylogenetic relationship among the Ago/Piwi proteins of different species. While numerous miAgo (blue), phPiwi (lime green), and rePiwi (pastel green) were identified in Mollusca, siAgo (yellow) was only identified in outgroup reference species: *D. melanogaster*, *B. morii*, *S. mediterranea*, *T. castaneum*, and *C. elegans*.** B** Overview of phylogenetic relationship among the Dicer proteins of different species. While numerous miRNA (blue) were identified in Mollusca, siDicer was only identified in the outgroup reference species. **C** Comparison of siDicer-like sequences present in *D. melanogaster,* which has endogenous siRNAs, *M. musculus*, which has few siRNAs, *S. mediterranea*, in which dsRNA-RNAi has been elicited, and *C. teleta*, which has no siRNAs, to select Mollusca species: *O. bimaculoides*, *M. gigas*, *C. virginica*, *M. yessoensis*, *H. rufescens*,* L. stagnalis*, and *R. venosa*. The outgroup *L*. *variegatus* was also analyzed. The percentage indicates the number of siDicer-like sequences compared to total filtered RNA-seq sequences per organism.

To assess available gene silencing pathways in mollusks, we characterized the small RNA biogenesis factors, Dicer and Ago/Piwi proteins, in 35 genomes (Fig. 1, Sup. Table 1). Using annotations of genomes in NCBI and Ensembl Metazoa along with BLAST searches, putative homologs of Ago/Piwi and Dicer proteins were identified (Sup. File 1, Sup. File 2). Next, using the MEGA 12 software package, multi-sequence alignments of peptides were generated to predict function through homology to well-characterized RNAi factors. Orthologs from *C. teleta*,* T. castaneum*, *B. mori*, *D. melanogaster*,* C. elegans*, *L. variegatus*,* S. mediterranea*, and *H. sapiens* were also included (Fig. 1A-B, Sup. Figures 1–2). These species were chosen due to either the extensive characterization of their RNAi factors or their position on the animal phylogenetic tree (Elbashir et al. 2001; Yamaguchi et al. 2011; Gu et al. 2012; Wang et al. 2013). Importantly, insects and flatworms (*T. castaneum*, *B. mori*, *D. melanogaster*, and *S. mediterranea)* have siAgo and siDicer. In all mollusks, miRNA and piRNA biogenesis factors were clearly identified, which was not the case for those dedicated for siRNA biogenesis (Sup. Figure 3).

All mollusks have at least one miAgo, with several examples of group-specific expansion (Fig. 1A, Sup. Figure 1). For example, all oyster species have two miAgos that appear to be the result of duplication before their diversification. A similar situation is seen with Octopoda. There are some additional cryptic duplications seen in snails and clams. Branch lengths in the siAgo clades are very short, suggesting strong sequence conservation, as is typical for these proteins (Flynt 2021). Greater differences were seen in the Piwi homologs, specifically phPiwi class proteins, with duplications that occurred early in the diversification of groups. Many species have a single phPiwi; however, some bivalves, abalones, and snails have additional members that have significantly diverged sequences. Apart from the limpet *P. vulgata*, which has three phPiwis, only one additional phPiwi was observed where there was duplication. rePiwis, on the other hand, were found as single copies in all mollusk genomes. Overall, the relatedness of different Ago/Piwi proteins followed the phylogenetics of the clade. One exception was *G. aegis*, which likely has an accelerated rate of divergence due to extremophile adaptation. While miAgo has seen duplication, miDicer is more static, with all mollusks having only a single Dicer protein (Fig. 1B, Sup. Figure 2). The relatedness of miDicer proteins also followed the phylogenetics of the clade, with a distinct divergence between the bivalves and gastropods, and a greater divergence between mollusk miDicers and outgroups. No mollusk siDicer-like proteins were identified that cluster with insect siDicers, where long branch lengths were observed, consistent with rapid evolution (Felsenstein 1978; Kling et al. 2018).

To further investigate the predicted RNAi pathways in Mollusca, we performed small RNA sequencing on samples from *C. virginica* gonad, muscle, gill, and whole juvenile animals, along with muscular tissue from *H. rufescens*, tentacle tissue from *O. bimaculoides*, and gonadal tissue from the echinoderm outgroup *L. variegatus*. Public sRNA-seq data from *M. gigas*, *M. yessoensis*, *L. stagnalis*, and *R. venosa* were also included to represent groups in the phylum (Sup. Figure 4, Sup. Table 2). In total, ~ 2.3B small reads were analyzed, with mapping rates ranging from ~ 63% to ~ 98%. Using alignments of these datasets, the presence of an siRNA pathway was examined using an algorithm that finds pairs of reads that overlap in alignments that are ~ 20–24 nt and also have 2 nt overhangs (Mondal et al. 2018). Such read pairs represent potential Dicer products due to their similarities to RNAs cleaved from dsRNAs. The number of these potential Dicer pairs was then divided by the total alignments from the library to calculate the percent of Dicer pairs. For reference, this was also calculated for *D. melanogaster* and *S. mediterranea*, which have endogenous siRNAs, and *Mus musculus*, which in rare settings produces endo-siRNA-like small RNAs (Ghildiyal et al. 2008; Rouhana et al. 2013; Garcia-Lopez et al. 2015). As a negative control, we included *C. teleta*, which has no known siRNAs (Fig. 1C) (Khanal et al. 2022). In species with siRNAs, 14.4%–0.05% siRNA-like sequences were found; in sharp contrast, each mollusk species’ results were at a minimum fivefold lower, showing similarity to *C. teleta*. Our findings reinforce the observation that siRNAs are not made in mollusk cells. This is consistent with loss of siRNA machinery occurring before the split with annelids (Khanal et al. 2022).

To understand if miRNA biology has also changed in mollusks, we used the miRDeep2 software to annotate and document conservation patterns with the sequencing datasets (Sup. Table 3) (Friedlander et al. 2012). Many conserved miRNA families were identified across the clade, with mir-2 and mir-92 having the highest number of duplications. Numerous putative novel miRNAs were also noted in our survey (Sup. Files 3–9). Together, this shows that miRNA function has not significantly changed in mollusks, consistent with conservation patterns of miAgo and miDicer.

### Mollusk sRNA Population Survey

**Fig. 2:**
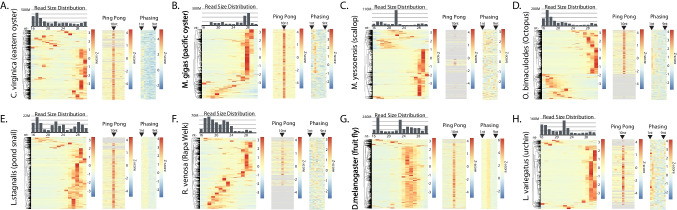
Small RNA species in mollusks. Size distribution of small RNA and piRNA processing signatures in small RNA alignments found in **A** *C. virginica* (~ 5800 sRNA-producing loci),** B** *M. gigas* (~ 5400 loci),** C** *M. yessoensis* (~ 3800 loci),** D** *O. bimaculoides* (~ 5700 loci),** E** *L. stagnalis* (~ 5900 loci),** F** *R. venosa* (~ 4800 loci), **G** *D. melanogaster* (~ 7800 loci), and **H** the outgroup *L. variegatus* (~ 3500 loci). Each bar graph shows bulk reads across all the species’ libraries. Each heatmap (row normalized) shows the sRNA size distribution per loci. Ping Pong signature shows 10 nt overlap expression. Phasing signature is based on the distance of 1U reads to an upstream alignment. Positions 1 to 9 from the upstream alignment are shown. Order of loci in each heatmap are preserved from clustering of size distribution.

Next, to further inform gene silencing approaches in mollusks, we characterized endogenous sRNAs of 8 species for which we have sequencing data (Fig. 2, Sup. Table 2). First, the size distribution of all reads aligning to genomes was determined. This allows differentiation between Dicer products (miRNAs and endo-siRNAs) that are 18–24 nt long and piRNAs, which are 26–30nt. For all mollusks, two peaks were seen that represent these two different types of small RNA (Fig. 2A-F). As a comparison, this was also performed on libraries from *D. melanogaster* gonads and tissue from *L. variegatus* (urchin), which show the same two peaks (Fig. 2G, H). The relative portion of Dicer-produced small RNAs to piRNAs varies by species, suggesting divergent roles for the pathways; however, some of this variability might be attributable to libraries being created from different tissues. Some of the datasets (i.e., *C. virginica*, *L. stagnalis*) show a peak below 18 nts, which likely represents degradation products, further suggesting that inter-library differences might contribute to the distinct contours of the size distributions. Nevertheless, it is clear that all the mollusks as well as urchins have somatic piRNAs.

To further investigate piRNAs, we annotated small RNA-expressing loci using small read depth and merging adjacent features (Khanal et al. 2022). Due to the differences in sequencing depth, appropriate thresholds were established from *C. virginica* data (Sup. Figure 5). By merging nearby features, accidentally splitting clusters into multiple candidate loci can be avoided. Similarly, setting a minimum read depth avoids inclusion of low confidence loci. For this analysis, *C. virginica* was used since it has a chromosome-level genome assembly and is the species for which we have the greatest volume of small RNA-seq data. To determine the threshold, regions of interest were combined when within either 0, 5, 50, 500, 5000, or 50,000 bp of one another. The subsequent loci of these mergers were then filtered for loci with a minimum read depth ranging from 0 to 1 million reads. We concluded that optimal regions of interest that were identified from merging adjacent features within 500 bp and had a coverage of  ≥ 1000 RPM (Sup. Figure 5A). These parameters yielded reasonably specific coverage of the *C. virginica* genome at 0.36% (Sup. Figure 5B). In *C. virginica*, this methodology identified ~ 5800 loci-expressing sRNAs (Sup. File 7). These thresholds were used to identify sRNA regions in other species (Sup. Files 10–17).

Using the optimized annotations, we assessed the read size distribution at individual loci in the species to assess abundance of loci generating different sRNA classes (Fig. 2). To visualize loci, values were Z-normalized and plotted as rows of a hierarchically sorted heatmap. For all species, including urchin and fruit fly,  > 50% of the loci had a bias for piRNA-sized reads. miRNA loci, in contrast appear to be a minority. Consistent with the overall size distributions, some species, such as *R. venosa*, had many loci called by mapping of putative degradation products. To further validate piRNA candidate loci, Ping Pong and phasing were assessed through calculating overlap bias and distance between reads, respectively (Han et al. 2015). At least some Ping Pong alignments were seen in all species at loci that also showed bias for piRNA-sized reads. Similarly, phasing was detected at potential piRNA loci with the exception of *M. yessoensis* (Fig. 2H). This is likely the result of a poorly assembled genome. For example, while *C. virginica*’s genome assembly consists of only 11 contigs, *M. yessoensis*’s assembly consists of 82,659. The presence of abundant apparent piRNA-expressing loci in Mollusca is similar to observations in *C. teleta*. This further reinforces the observation that mollusks and annelids share RNAi biology.

### Localization of C. virginica piRNAs

**Fig. 3:**
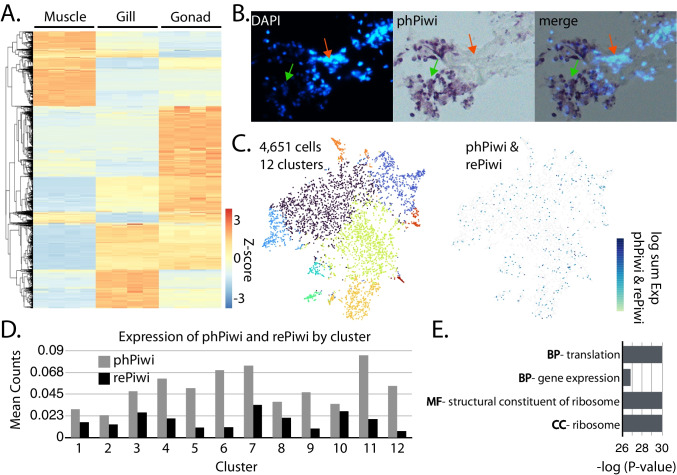
piRNA and Piwi expression in *C. virginica* tissues. **A** Row normalized heatmap showing piRNA (26–30 nt) expression at putative piRNA loci in *C. virginica* muscle, gill, and gonad tissue. **B** An overlay of DAPI staining of nuclei and colorimetric visualization of Piwi via in situ hybridization. **C** tSNA plot showing clustering of 4651 cells into twelve clusters (left) from *C. virginica* mantle tissue. Detection of cells that express phPiwi and/or rePiwi found in the cell population*.* **D** Mean counts of phPiwi and rePiwi in the different cell clusters. **E** GO analysis of genes differentially expressed in phPiwi and rePiwi expressing cells. BP, biological process; MF, molecular function; CC, cellular component.

Most mollusks appear to have abundant piRNAs in both soma and germline, suggesting that piRNA-based gene silencing may be possible, as this has been achieved in other animals with somatic piRNAs (Mondal et al. 2020). To explore this possibility, we examined the expression of Piwi in tissues of *C. virginica* (Fig. 3). Eastern oysters are often raised in aquaculture and therefore are a good candidate for biotechnology (Vilsack 2024). We first sought to determine if similar piRNA expression was observed in both somatic and germline tissue. To accomplish this, we compared piRNA expression using samples from muscle, gill, and gonadal tissue. Selecting for only piRNA-like loci based on both their size distribution and high Ping Pong z-score, we compared loci expression in each tissue type (Fig. 3A). Our comparison revealed distinct piRNA pathways in each of the tissue types, with some shared expression between the gill and gonadal tissue.

While all tissues expressed piRNAs, the variability between the tissues raised the question of which cell types generate piRNAs. To resolve if piRNAs are expressed broadly throughout tissues or if they are only present in subsets, we localized expression of *C. virginica* phPiwi using in situ hybridization on tissue from the mantle (Fig. 3B, Sup. Figure 6). From this, we found that not all cells were stained for phPiwi, indicating that piRNA expression is likely restricted. This observation suggested that though piRNAs are present in each tissue, they are only present in certain cell types; thus, a clearer understanding of cell identities would be needed for piRNA-based gene silencing to be realized. To characterize the identities of Piwi expressing cells, we pioneered single-nuclei RNA sequencing (snRNA-seq) in oysters using mantle tissue (Fig. 3C). This experiment led to recovery of 4651 cells which clustered into 12 groups. Oyster mantle has been described on the histological level, but not at all on the cellular (Zúñiga-Soto et al. 2023). This makes it challenging to understand the identities of cells; however, when searching for *C. virginica* phPiwi and rePiwi transcripts within the clusters found, they did not map specifically to any group. Looking at counts of the phPiwi and rePiwi within the clusters, we found some variability, with phPiwi having a higher expression level, though transcripts were clearly found in all (Fig. 3D). Together, this shows that Piwi expressing cells are not a well-defined cell type in a tissue, but rather a subset of many cell identities.

To understand the unique features of Piwi-expressing cells, differential expression was performed for these cells relative to the overall population. Roughly 90 genes were found to be differentially expressed, nearly all of which were downregulated in the Piwi cells. GO analysis was then performed on the gene set, which showed highly significant enrichment for genes involved in translation (Fig. 3E). Identities of the genes included both initiation factors and components of the ribosome itself. All these genes related to translation in the gene set were downregulated, suggesting that Piwi expression co-occurs with quiescence. Such a state is observed in stem cells (Tahmasebi et al. 2018). If the piwi-positive cells are in fact stem cells within each cell type, this would align with observations in *C. teleta* (Giani et al. 2011). These stem cells may be involved in regeneration and maintenance of the tissue. Alternatively, the cells could be experiencing a state of stress, and Piwi expression is a component of that response (Advani and Ivanov 2019).

### Role of piRNAs in Mollusk Regeneration

**Fig. 4:**
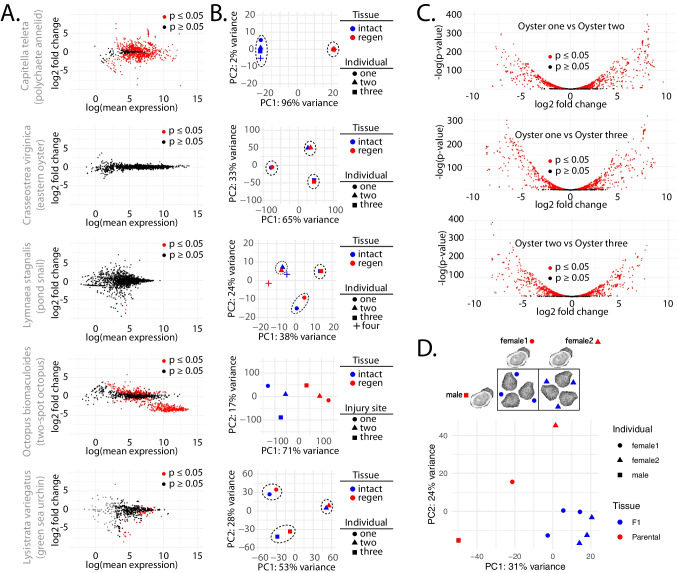
piRNA expression during regeneration and comparison of expression between parental and F1 animals. **A** Scatterplots showing piRNA differential expression in uninjured tissue and regenerating tissue in *C. teleta*, *C. virginica*, *L. stagnalis*, *O. bimaculoides*, and *L. variegatus*. Black dots are not significant (*p* value ≥ 0.05), while red dots are significant (*p* value ≤ 0.05). **B** PCA of piRNA expression in each species’ individual intact (blue) and regenerating tissues (red). Dashed lines highlight closely clustering individuals. **C** Volcano plots showing the differential expression of piRNAs between datasets from individual animals. Black dots are not significant (*p* value ≥ 0.05), while red dots are significant (*p* value ≤ 0.05). **D** PCA of piRNA expression in parents and F1 eastern oysters. Breeding scheme shown above where sperm from a single male (square) was used to fertilize eggs from two different females (circle and triangle) to yield two sets of juvenile oysters. Relation of juvenile oysters to which mother is shown by matching shape of the data point (circle and triangle).

Work in *C. teleta* demonstrated that Piwi expression is present in regenerating tissues and other proliferative cells, and the work here in oysters suggests a similar biology (Giani et al. 2011). To investigate if piRNAs have a consistent function that relates to regeneration, we investigated piRNA expression in intact and regenerating tissues in *C. teleta*,* C. virginica*,* L. stagnalis*,* O. bimaculoides*,and* L. variegatus* (Fig. 4A, B). For each species, somatic tissues were harvested (Sup. Table 2). All data, excluding those from *L. stagnalis* (Bioproject PRJNA664475), were generated as part of this study. Consistent with observations in *C. teleta*, we found significant changes in piRNAs (annotated in prior work) expressed in intact vs regenerating tissues in this annelid (Fig. 4A). Comparison of individual samples revealed high similarity between the tissue types (Fig. 4B). Unexpectedly, an opposite outcome was seen when investigating differential expression of oyster piRNAs. Only a single piRNA locus showed a significant difference in expression being upregulated by ~ 1.4 log2 fold change. The difference extended to clustering via PCA, where samples from the same individual showed more similarity than tissue types (Fig. 4B). This situation was also seen in *L. stagnalis*, with only a handful of differentially expressed piRNA loci. Unfortunately, the public dataset metadata did not indicate whether intact and regenerating samples were from the same individual; however, based on sample clustering, some may have been acquired from the same animal. *O. bimaculoides* data was more similar to *C. teleta*, with many significantly differentially expressed piRNAs. Unlike other species examined here, samples were collected from a single individual at different sites (tentacle tips). As part of this study, we also examined piRNA expression during regeneration in *L. variegatus* as an outgroup. Interestingly, the urchin data showed similarity to *C. virginica* and *L. stagnalis*. This suggests that the highly individualized piRNA expression that we see in oysters and snails might be the ancestral state, and that the situation in *C. teleta* is derived. The octopus data suggest that piRNA expression may likely change during regeneration in mollusks; however, individual differences in piRNA abundance might exceed the changes that occur in response to tissue damage.

When comparing the differences in expression between the individual oysters—comparing intact and regenerating samples from one oyster to another—we found highly divergent patterns of piRNA expression (Fig. 4C). These animals were all raised in a single hatchery from the same brood stock. Despite this, the number of loci showing differential expression exceeded 55% of the total number of piRNA annotations. To investigate the origin of the disparate piRNA expression seen between animals, sperm from one male was used to fertilize eggs from two females, collecting both parental and F1 tissues (Fig. 4D). Here, one Male oyster was used with two females. Offspring were then allowed to mature for 3 months to a juvenile stage. The breeding scheme was designed so that if F1s showed similarity to a parent, this would indicate the basis of an individual’s piRNA configuration. When comparing variance in piRNA expression via PCA, we found the juveniles cluster together, while the parents were quite distant from their offspring and each other. On the PC2 axis, offspring showed closeness to the father, while both mothers were further from their respective children. This is a little surprising given that piRNAs have been found to be inherited maternally in an extra-nuclear fashion (Mani et al. 2014). These results suggest that the piRNA repertoire in oysters is contributed by the genetics of both parents, and that as animals age, expression may become increasingly divergent.

## Discussion

Exploration of mollusk small RNA pathways found many miRNAs and piRNAs, but no evidence of a dedicated endo-siRNA pathway. Mollusk piRNAs have a distinct biology relative to ecdysozoans and vertebrates with abundant somatic piRNAs that appear to be restricted to only a portion of cells within tissues. piRNA expression patterns are also highly individualized and are only partially defined by inheritance. Moreover, unlike other animals, it appears that piRNA expression is determined equally by maternal and paternal inheritance. Together, these observations have implications for gene silencing approaches, which rely on exploiting an endogenous mechanism, in mollusks.

Mollusk fisheries in the United States face significant challenges, highlighted by a 93% decline in eastern oyster landings due to the collapse of wild harvesting (Haven et al. 1978; Pelton and Goldsborough 2010; Speights et al. 2017; MacKenzie and Tarnowski 2018; La Peyre et al. 2020; Pruett et al. 2022; Wontor et al. 2023). To combat this decline, aquaculture has become the predominant method of propagating many species (Vilsack 2024). This provides an opportunity for biotechnology to induce desirable traits such as robustness to environmental stress or disease resistance. This does raise some ethical and environmental concerns with modified animals being placed in the environment to fully mature. Many oyster hatcheries produce triploid offspring, which are considered sterile and therefore have a reduced concern for spread of modified alleles or transgenes into wild populations (He et al. 2022; Nwokeoji et al. 2022). Many challenges exist for these approaches, including isolation of genetically modified brood stock from wild populations or delivery of large molecules such as Cas9-gRNA complexes in aquaculture facilities. These limitations could be addressed with RNAi technology, as it does not lead to permanent genomic changes and can be applied with large-scale RNA synthesis to oysters during early developmental stages while they are being raised in large volumes of seawater (He et al. 2022; Nwokeoji et al. 2022; Wenninger et al. 2025).

Gene silencing in invertebrates is typically triggered by delivery of long dsRNA to exploit the anti-viral or endogenous siRNA pathway (Zhang and Ruvkun 2012; Haiyong 2018). Here we show that mollusks, and possibly the entire Lophotrochozoan clade, do not appear to have a dedicated siRNA pathway that would process long dsRNA into siRNAs. The apparent absence of endo-siRNAs could be an issue of the sensitivity of detection. In mice, for instance, long dsRNA is processively processed into siRNA-like sRNAs, but only in oocytes by a specific dicer isoform (Flemr et al. 2013). These siRNA-like molecules load into miAgo and are not 2’O methylated, distinguishing them from the siRNAs generated in ecdysozoans. Nevertheless, it is possible that mollusks may process endogenous long dsRNA in rare cell types or under specific circumstances, such as stress or infection. However, our *C. virginica* and public *M. gigas* datasets include samples from various gonadal and somatic tissues and would be predicted to include rare siRNAs like those reported in mouse oocytes. Importantly, if endo-siRNAs are present but only at a low level, it argues against using long dsRNA for gene silencing. A robust technology would operate in all cells. It is also possible that mollusk siRNAs have been chemically modified such that they are not captured during library preparation (Shi et al. 2021). In the datasets published in this article, many piRNAs are captured, which are modified sRNAs (Pastore et al. 2021). Even more compelling is that small RNA modifications that define their type happens after loading into Ago/Piwi proteins, suggesting that mollusks with only one or two Ago orthologs (Fig. 1) would have limited options for segregating a population of sRNAs to target for modification.

To date, there have been many reports of using long dsRNA for gene silencing in mollusks as presented and referenced above in the introduction. Unlike vertebrates, many mysteries remain regarding the cellular response to long dsRNA molecules in the mollusk cells. For example, it has not been reported if long dsRNA is recognized as a pathogen pattern. However, based on the fact that planarians respond to long dsRNA with induction of apoptosis, this suggests that at a minimum this molecule may be recognized as pathogenic in higher spiralians (Kozlovski et al. 2024). Another consideration is that mollusks have highly repetitive genomes, providing many opportunities for production of dsRNA from bidirectional transcription or transcription of inverted repeats. Despite this, evidence of long dsRNA processing by Dicer was elusive in our analysis, suggesting that other mechanisms may be present, such as RNA editing to melt dsRNAs (Liu et al. 2024). Based on these observations, reported gene silencing by long dsRNAs may be the result of distributive processing of dsRNA where the ends of molecules are cleaved by Dicer. Further investigation is needed to understand the fate of long dsRNA; however, if Dicer does generate small RNAs from these molecules, they will be loaded into miAgo proteins. This suggests that the design of RNAi reagents may be more appropriate to follow practices from vertebrate systems where small duplex RNAs or short-hairpin RNAs (shRNA) are used to mimic miRNAs (LaMonte et al. 2012; Cyrus 2021; Kim and Croce 2023). Fundamentally, commercialized gene silencing technologies that use long dsRNA are employed in animals where endo-siRNAs can be readily detected (Reinders et al. 2023). In animals where endo-siRNAs are not detected, or found only in a minority of cells, small duplex RNA and shRNA are used (Adams et al. 2018).

In contrast to endo-siRNAs, many piRNAs are present in mollusks. Though production of ectopic piRNAs in somatic tissues has been demonstrated in insects for gene silencing, such an approach would also not be appropriate in mollusks (Mondal et al. 2020). In this study, we observe that Piwi proteins are not present in all cells within tissues. (Fig. 3B-C). Gene expression profiles in Piwi-expressing cells suggest that these are likely stem cells where piRNAs may have a role in maintaining genome integrity, possibly through suppressing transposable elements (Toth et al. 2016; Luo and Lu 2017). Similar stem cell restriction of Piwi and piRNAs has been observed in *C. teleta* and planarians (Giani et al. 2011; Rink 2013; Kim et al. 2018). Thus, mollusks seem to share a role for piRNAs with members of the larger spiralian clade. This lack of ubiquitous piRNAs in tissues raises concerns about the usefulness of piRNA-mediated RNAi in *C. virginica* and likely the broader Mollusca phylum. A robust gene silencing technology would ideally operate in all cell types. Furthermore, we find that individuals of some mollusk species have a distinct set of piRNAs. Gene silencing by piRNA requires identification of a sequence that can recruit the phasing piRNA machinery. In animals with individualized piRNA profiles, a common sequence cannot be found, meaning silencing may occur in one animal but not another. Thus, as mentioned above, the most viable means of inducing RNAi in mollusks will likely be through exploiting the miRNA pathway using small duplex RNAs or shRNAs.

In addition to informing technology development, the results of this study also provide insights into the fundamental biology of gene expression in mollusks. Studies have found that on the population level, oyster genomes are highly polymorphic (Li et al. 2021). This is likely a consequence of the need for sessile animals, such as oysters, to be phenotypically plastic and respond to environmental conditions. Indeed, shell formation is a complex process that generates varying external morphologies (e.g., size, shape) (Launey and Hedgecock 2001). The increased genetic variability in these animals also seems to lead to proliferation of deleterious alleles. Investigation of the Pacific oyster genome found evidence for non-Mendelian inheritance that likely compensates for a dominant high genetic load (Launey and Hedgecock 2001). Additionally, heterosis has been observed in oysters, suggesting significant individual genetic differences (Hedgecock and Davis 2007). Other studies have found significant chromosomal abnormalities accumulating in oyster tissues, suggesting the negative alleles are also accompanied by genomic instability (Zenaba et al. 2022). This situation informs the likely role and abundance of piRNAs in oysters. By expressing these RNAs in a stem cell-like compartment, better genome integrity in this cell type could help preserve cell viability during growth or regeneration. Bivalve genomes seem to be routinely remodeled by transposons (Martelossi et al. 2023). The expansion of parasitic genetic parasites could be the basis of genetic variability seen in oysters and reinforces the importance of piRNAs in balancing transposon activity throughout the animal’s tissues.

## Supplementary Information

Below is the link to the electronic supplementary material.

ESM 1 (ZIP 581 KB)

## Data Availability

No datasets were generated or analysed during the current study.
